# (*Z*)-2-[(2-Hydr­oxy-1-naphth­yl)methyl­eneamino]benzonitrile

**DOI:** 10.1107/S1600536809023708

**Published:** 2009-06-27

**Authors:** Jian-Cheng Zhou, Chuan-Ming Zhang, Nai-Xu Li, Zheng-Yun Zhang

**Affiliations:** a College of Chemistry and Chemical Engineering, Southeast University, Nanjing 211189, People’s Republic of China

## Abstract

The title compound, C_18_H_12_N_2_O, crystallizes in a phenol–imine tautomeric form with a *Z* conformation for the imine functionality. The dihedral angle between the aromatic rings is 8.98 (9)°. A strong intra­molecular O—H⋯N hydrogen-bond inter­action between the hydroxyl group and imine N atom occurs.

## Related literature

For general properties of Schiff base compounds, see: Weber *et al.* (2007 ▶); Chen *et al.* (2008 ▶). For related structures, see: Elmali *et al.* (2001 ▶); Yüce *et al.* (2006 ▶); Petek *et al.* (2007 ▶).
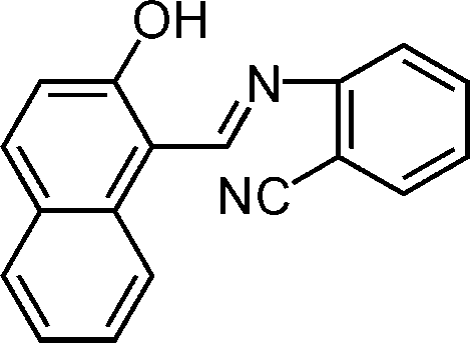


## Experimental

### 

#### Crystal data

C_18_H_12_N_2_O
*M* *_r_* = 272.30
Monoclinic, 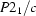

*a* = 13.4640 (13) Å
*b* = 7.4450 (6) Å
*c* = 15.4090 (11) Å
β = 116.660 (6)°
*V* = 1380.4 (2) Å^3^
*Z* = 4
Mo *K*α radiation
μ = 0.08 mm^−1^
*T* = 293 K
0.20 × 0.20 × 0.20 mm

#### Data collection

Rigaku SCXmini diffractometer
Absorption correction: multi-scan (*CrystalClear*; Rigaku, 2005 ▶) *T*_min_ = 0.973, *T*_max_ = 0.979
12133 measured reflections
2706 independent reflections
1803 reflections with *I* > 2σ(*I*)
*R*_int_ = 0.056

#### Refinement

*R*[*F* ^2^ > 2σ(*F* ^2^)] = 0.066
*wR*(*F* ^2^) = 0.159
*S* = 1.10
2706 reflections
190 parameters
H-atom parameters constrained
Δρ_max_ = 0.20 e Å^−3^
Δρ_min_ = −0.18 e Å^−3^

### 

Data collection: *CrystalClear* (Rigaku, 2005 ▶); cell refinement: *CrystalClear*; data reduction: *CrystalClear*; program(s) used to solve structure: *SHELXS97* (Sheldrick, 2008 ▶); program(s) used to refine structure: *SHELXL97* (Sheldrick, 2008 ▶); molecular graphics: *SHELXTL* (Sheldrick, 2008 ▶); software used to prepare material for publication: *SHELXL97*.

## Supplementary Material

Crystal structure: contains datablocks I, global. DOI: 10.1107/S1600536809023708/bh2232sup1.cif

Structure factors: contains datablocks I. DOI: 10.1107/S1600536809023708/bh2232Isup2.hkl

Additional supplementary materials:  crystallographic information; 3D view; checkCIF report

## Figures and Tables

**Table 1:** Hydrogen-bond geometry (Å, °)

*D*—H⋯*A*	*D*—H	H⋯*A*	*D*⋯*A*	*D*—H⋯*A*
O1—H1*A*⋯N1	0.82	1.82	2.551 (2)	147

**Fig. 1.**
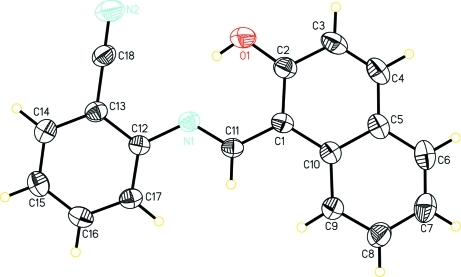
The molecular structure of the title compound, showing the atomic numbering scheme. Displacement ellipsoids are drawn at the 30% probability level.

## supplementary crystallographic information

### Comment

Schiff base compounds have received considerable attention for many years because these compounds play an important role in coordination chemistry related to magnetism (Weber *et al.*, 2007) and catalysis (Chen *et al.*, 2008).Our group is interested in the synthesis and preparation of Schiff bases. Here, we report the synthesis and crystal structure of the title compound.

Figure 1 shows an *ORTEP* plot of the title compound. The molecule adopts the phenol–imine tautomeric form with a strong intramolecular O—H···N hydrogen bond. The C11N1 and C2—O1 bond lengths [1.296 (3) and 1.324 (3) Å, respectively] are comparable to corresponding values observed in a similar phenol–imine tautomeric structures (*e.g*. Petek *et al.*, 2007), while different geometry is observed in the case of zwitterionic molecules (Elmali *et al.*, 2001; Yüce *et al.*, 2006). Phenyl and naphthalyl rings, *A* (C12···C17) and *B* (C1···C10), are, of course, planar, and the dihedral angle between them is 8.98 (9)°. The molecule displays a *trans* configuration about the central CN imine bond. Molecules are packed in the crystal at van der Waals distances.

### Experimental

2-Aminobenzonitrile (0.59 g, 5 mmol) and 2-hydroxynaphthalene-1-carbaldehyde (0.861 g, 5 mmol) were dissolved in ethanol (25 ml). The resulting mixture was refluxed for 5 h and cooled to room temperature. The solid product was collected by filtration. Crystals suitable for X-ray diffraction studies were obtained on slow evaporation at room temperature.

### Refinement

The H atoms were placed geometrically and treated as riding atoms with O—H = 0.82 Å and C—H = 0.93 Å, and with *U*_iso_(H) = 1.2*U*_eq_(Carrier C) and *U*_iso_(H1A) = 1.5*U*_eq_(O1).

### Crystal data

**Table:**

C_18_H_12_N_2_O	*F*(000) = 568
*M**_r_* = 272.30	*D*_x_ = 1.310 Mg m^−^^3^
Monoclinic, *P*2_1_/*c*	Mo *K*α radiation, λ = 0.71073 Å
Hall symbol: -P 2ybc	Cell parameters from 2198 reflections
*a* = 13.4640 (13) Å	θ = 2.7–27.5°
*b* = 7.4450 (6) Å	µ = 0.08 mm^−^^1^
*c* = 15.4090 (11) Å	*T* = 293 K
β = 116.660 (6)°	Block, pale yellow
*V* = 1380.4 (2) Å^3^	0.20 × 0.20 × 0.20 mm
*Z* = 4	

### Data collection

**Table:**

Rigaku SCXmini diffractometer	2706 independent reflections
Radiation source: fine-focus sealed tube	1803 reflections with *I* > 2σ(*I*)
graphite	*R*_int_ = 0.056
Detector resolution: 13.6612 pixels mm^-1^	θ_max_ = 26.0°, θ_min_ = 3.0°
ω scans	*h* = −16→16
Absorption correction: multi-scan (*CrystalClear*; Rigaku, 2005)	*k* = −9→9
*T*_min_ = 0.973, *T*_max_ = 0.979	*l* = −18→18
12133 measured reflections	

### Refinement

**Table:**

Refinement on *F*^2^	Primary atom site location: structure-invariant direct methods
Least-squares matrix: full	Secondary atom site location: difference Fourier map
*R*[*F*^2^ > 2σ(*F*^2^)] = 0.066	Hydrogen site location: inferred from neighbouring sites
*wR*(*F*^2^) = 0.159	H-atom parameters constrained
*S* = 1.10	*w* = 1/[σ^2^(*F*_o_^2^) + (0.0672*P*)^2^ + 0.1372*P*] where *P* = (*F*_o_^2^ + 2*F*_c_^2^)/3
2706 reflections	(Δ/σ)_max_ < 0.001
190 parameters	Δρ_max_ = 0.20 e Å^−^^3^
0 restraints	Δρ_min_ = −0.18 e Å^−^^3^
0 constraints	

### Fractional atomic coordinates and isotropic or equivalent isotropic displacement parameters (Å^2^)

**Table:**

	*x*	*y*	*z*	*U*_iso_*/*U*_eq_	
O1	0.22890 (15)	0.5907 (3)	−0.07648 (12)	0.0792 (6)	
H1A	0.1718	0.6436	−0.0852	0.119*	
N1	0.07487 (14)	0.7038 (2)	−0.03712 (12)	0.0490 (5)	
N2	0.0188 (2)	0.7966 (4)	−0.27347 (16)	0.0894 (8)	
C1	0.24373 (17)	0.5812 (3)	0.08450 (16)	0.0465 (5)	
C11	0.13677 (17)	0.6610 (3)	0.05282 (16)	0.0459 (5)	
H11A	0.1105	0.6831	0.0983	0.055*	
C12	−0.03185 (17)	0.7809 (3)	−0.07090 (15)	0.0439 (5)	
C13	−0.08320 (18)	0.8360 (3)	−0.16755 (16)	0.0500 (6)	
C10	0.30954 (17)	0.5267 (3)	0.18411 (16)	0.0477 (6)	
C6	0.4729 (2)	0.3710 (3)	0.3064 (2)	0.0674 (7)	
H6A	0.5381	0.3068	0.3228	0.081*	
C5	0.41020 (19)	0.4302 (3)	0.21022 (18)	0.0550 (6)	
C9	0.2793 (2)	0.5638 (3)	0.25909 (17)	0.0582 (6)	
H9A	0.2150	0.6290	0.2451	0.070*	
C2	0.2839 (2)	0.5440 (3)	0.01600 (18)	0.0569 (6)	
C17	−0.08801 (19)	0.8058 (3)	−0.01468 (16)	0.0528 (6)	
H17A	−0.0552	0.7709	0.0502	0.063*	
C16	−0.1922 (2)	0.8823 (3)	−0.05539 (18)	0.0579 (6)	
H16A	−0.2292	0.8983	−0.0175	0.069*	
C14	−0.1883 (2)	0.9135 (3)	−0.20763 (18)	0.0596 (7)	
H14A	−0.2216	0.9502	−0.2722	0.072*	
C15	−0.2426 (2)	0.9355 (3)	−0.15123 (19)	0.0605 (7)	
H15A	−0.3133	0.9862	−0.1776	0.073*	
C4	0.4449 (2)	0.3949 (3)	0.1372 (2)	0.0664 (7)	
H4A	0.5105	0.3316	0.1537	0.080*	
C3	0.3859 (2)	0.4500 (4)	0.0453 (2)	0.0684 (8)	
H3A	0.4121	0.4264	−0.0001	0.082*	
C18	−0.0262 (2)	0.8130 (4)	−0.22658 (17)	0.0624 (7)	
C8	0.3432 (2)	0.5053 (4)	0.35194 (19)	0.0716 (8)	
H8A	0.3218	0.5322	0.4001	0.086*	
C7	0.4394 (2)	0.4064 (4)	0.3755 (2)	0.0749 (8)	
H7A	0.4808	0.3647	0.4386	0.090*	

### Atomic displacement parameters (Å^2^)

**Table:**

	*U*^11^	*U*^22^	*U*^33^	*U*^12^	*U*^13^	*U*^23^
O1	0.0708 (12)	0.1177 (17)	0.0570 (11)	0.0071 (11)	0.0359 (10)	−0.0072 (11)
N1	0.0484 (11)	0.0551 (12)	0.0439 (11)	−0.0048 (9)	0.0211 (9)	−0.0043 (9)
N2	0.0851 (17)	0.134 (2)	0.0598 (15)	0.0022 (16)	0.0421 (14)	0.0019 (14)
C1	0.0461 (12)	0.0447 (12)	0.0530 (14)	−0.0078 (10)	0.0259 (11)	−0.0084 (10)
C11	0.0498 (13)	0.0447 (12)	0.0473 (13)	−0.0059 (10)	0.0256 (11)	−0.0057 (10)
C12	0.0428 (12)	0.0450 (12)	0.0449 (12)	−0.0070 (10)	0.0205 (10)	−0.0072 (10)
C13	0.0498 (13)	0.0542 (14)	0.0475 (13)	−0.0075 (11)	0.0232 (11)	−0.0025 (11)
C10	0.0461 (12)	0.0406 (12)	0.0559 (14)	−0.0069 (10)	0.0224 (11)	−0.0025 (10)
C6	0.0505 (14)	0.0529 (15)	0.083 (2)	0.0003 (12)	0.0163 (15)	0.0074 (14)
C5	0.0471 (13)	0.0417 (12)	0.0716 (17)	−0.0036 (11)	0.0227 (13)	−0.0049 (12)
C9	0.0552 (14)	0.0652 (16)	0.0542 (15)	0.0035 (12)	0.0244 (12)	0.0035 (12)
C2	0.0525 (14)	0.0638 (16)	0.0570 (15)	−0.0058 (12)	0.0269 (12)	−0.0093 (12)
C17	0.0563 (14)	0.0571 (15)	0.0495 (13)	−0.0069 (12)	0.0278 (11)	−0.0025 (11)
C16	0.0577 (15)	0.0566 (15)	0.0701 (17)	−0.0052 (12)	0.0383 (13)	−0.0092 (13)
C14	0.0609 (15)	0.0595 (15)	0.0537 (15)	−0.0002 (12)	0.0214 (13)	0.0031 (12)
C15	0.0502 (14)	0.0540 (15)	0.0726 (18)	0.0010 (11)	0.0233 (13)	−0.0023 (13)
C4	0.0489 (14)	0.0572 (16)	0.092 (2)	0.0015 (12)	0.0304 (15)	−0.0130 (14)
C3	0.0612 (16)	0.0731 (19)	0.084 (2)	−0.0025 (14)	0.0443 (15)	−0.0200 (15)
C18	0.0610 (15)	0.0807 (19)	0.0448 (14)	−0.0019 (14)	0.0231 (12)	0.0029 (13)
C8	0.0692 (17)	0.085 (2)	0.0578 (16)	0.0000 (16)	0.0261 (14)	0.0081 (14)
C7	0.0678 (18)	0.0729 (19)	0.0686 (18)	0.0019 (14)	0.0169 (15)	0.0185 (15)

### Geometric parameters (Å, °)

**Table:**

O1—C2	1.324 (3)	C5—C4	1.423 (3)
O1—H1A	0.8200	C9—C8	1.368 (3)
N1—C11	1.296 (3)	C9—H9A	0.9300
N1—C12	1.412 (3)	C2—C3	1.423 (3)
N2—C18	1.139 (3)	C17—C16	1.377 (3)
C1—C2	1.412 (3)	C17—H17A	0.9300
C1—C11	1.426 (3)	C16—C15	1.378 (3)
C1—C10	1.444 (3)	C16—H16A	0.9300
C11—H11A	0.9300	C14—C15	1.374 (3)
C12—C17	1.394 (3)	C14—H14A	0.9300
C12—C13	1.393 (3)	C15—H15A	0.9300
C13—C14	1.390 (3)	C4—C3	1.340 (4)
C13—C18	1.439 (3)	C4—H4A	0.9300
C10—C9	1.414 (3)	C3—H3A	0.9300
C10—C5	1.423 (3)	C8—C7	1.390 (4)
C6—C7	1.355 (4)	C8—H8A	0.9300
C6—C5	1.408 (3)	C7—H7A	0.9300
C6—H6A	0.9300		
			
C2—O1—H1A	109.5	O1—C2—C3	117.6 (2)
C11—N1—C12	123.63 (18)	C1—C2—C3	119.9 (2)
C2—C1—C11	119.5 (2)	C16—C17—C12	119.8 (2)
C2—C1—C10	118.8 (2)	C16—C17—H17A	120.1
C11—C1—C10	121.64 (19)	C12—C17—H17A	120.1
N1—C11—C1	122.3 (2)	C15—C16—C17	121.3 (2)
N1—C11—H11A	118.8	C15—C16—H16A	119.4
C1—C11—H11A	118.8	C17—C16—H16A	119.4
C17—C12—C13	118.5 (2)	C15—C14—C13	119.6 (2)
C17—C12—N1	124.8 (2)	C15—C14—H14A	120.2
C13—C12—N1	116.66 (19)	C13—C14—H14A	120.2
C14—C13—C12	121.0 (2)	C14—C15—C16	119.8 (2)
C14—C13—C18	119.6 (2)	C14—C15—H15A	120.1
C12—C13—C18	119.4 (2)	C16—C15—H15A	120.1
C9—C10—C5	117.0 (2)	C3—C4—C5	122.0 (2)
C9—C10—C1	123.3 (2)	C3—C4—H4A	119.0
C5—C10—C1	119.6 (2)	C5—C4—H4A	119.0
C7—C6—C5	120.9 (3)	C4—C3—C2	121.0 (2)
C7—C6—H6A	119.6	C4—C3—H3A	119.5
C5—C6—H6A	119.6	C2—C3—H3A	119.5
C6—C5—C4	121.4 (2)	N2—C18—C13	179.3 (3)
C6—C5—C10	120.0 (2)	C9—C8—C7	121.1 (3)
C4—C5—C10	118.6 (2)	C9—C8—H8A	119.4
C8—C9—C10	121.1 (2)	C7—C8—H8A	119.4
C8—C9—H9A	119.5	C6—C7—C8	119.8 (3)
C10—C9—H9A	119.5	C6—C7—H7A	120.1
O1—C2—C1	122.4 (2)	C8—C7—H7A	120.1

### Hydrogen-bond geometry (Å, °)

**Table:**

*D*—H···*A*	*D*—H	H···*A*	*D*···*A*	*D*—H···*A*
O1—H1A···N1	0.82	1.82	2.551 (2)	147
